# Patient-adaptive Population-based Modeling of Arterial Input Functions

**DOI:** 10.1109/TMI.2022.3205940

**Published:** 2022-12-29

**Authors:** Zhaoyan Xiu, Mark Muzi, Jian Huang, Eric Wolsztynski

**Affiliations:** School of Mathematical Sciences, University College Cork, Ireland; School of Medicine, University of Washington, Seattle, WA 98195 USA; School of Mathematical Sciences, University College Cork, Ireland; School of Mathematical Sciences, University College Cork, Ireland and the Insight Centre for Data Analytics, Cork, Ireland

**Keywords:** Arterial input function estimation, kinetic modeling, medical imaging, parametric imaging, PET imaging

## Abstract

Kinetic modeling of dynamic PET data requires knowledge of tracer concentration in blood plasma, described by the arterial input function (AIF). Arterial blood sampling is the gold standard for AIF measurement, but is invasive and labour intensive. A number of methods have been proposed to accurately estimate the AIF directly from blood sampling and/or imaging data. Here we consider fitting a patient-adaptive mixture of historical population time course profiles to estimate individual AIFs. Travel time of a tracer atom from the injection site to the right ventricle of the heart is modeled as a realization from a Gamma distribution, and the time this atom spends in circulation before being sampled is represented by a subject-specific linear mixture of population profiles. These functions are estimated from independent population data. Individual AIFs are obtained by projection onto this basis of population profile components. The model incorporates knowledge of injection duration into the fit, allowing for varying injection protocols. Analyses of arterial sampling data from ^18^F-FDG, ^15^O-H_2_O and ^18^F-FLT clinical studies show that the proposed model can outperform reference techniques. The statistically significant gain achieved by using population data to train the basis components, instead of fitting these from the single individual sampling data, is measured on the FDG cohort. Kinetic analyses of simulated data demonstrate the reliability and potential benefit of this approach in estimating physiological parameters. These results are further supported by numerical simulations that demonstrate convergence and stability of the proposed technique under varying training population sizes and noise levels.

## I. Introduction

### A. Motivation

POSITRON Emission Tomography (PET) is widely used in the staging and evaluation of many diseases, and particularly in cancer. From the time of radiotracer injection, a PET scan provides 3D time course imaging of uptake of this radioactive agent in the body. Given the time course of radioactivity concentration in arterial plasma, called the Arterial Input Function (AIF), kinetic modeling of the dynamic imaging data can be used to measure physiologic parameters, such as blood flow and glucose metabolic rate [1]-[4]. There exist techniques for estimating physiological parameters that do not require the AIF, such as reference region approaches for receptor-radioligand studies [5]. To be valid, these techniques require the existence of a measurable, reliable reference region that is devoid of the target receptor, and for comparisons to be appropriate, the reference region properties must be independent of treatment effects and groups [5]. A region meeting these criteria has not yet been identified, or might not exist, for many radioligands. AIF estimation therefore constitutes a realistic, intermediate objective for many applications of dynamic data analysis for the determination of metabolic processes.

Determination of the AIF is a challenging task. The gold standard and most accurate approach is to use direct arterial blood sampling, but this requires multiple arterial blood samples over the whole scanning period, and presents several drawbacks including patient discomfort, additional costs, and potential risk due to its invasive nature. A number of less invasive methods have been considered that rely on one of a few strategies for arterial signal characterisation, as follows.

### B. Modeling of arterial input data

Image-derived input functions (IDIF) [6]-[13] are obtained by fitting an AIF model to arterial blood pool tracer activity extracted from PET image data, rather than obtained from arterial sampling. Such data are usually extracted from a region of interest (ROI) within a dynamic PET image that captures either a large blood vessel or the left cardiac ventricle, and therefore tend to be noisy. Usually, the average or the highest signals in the ROI are extracted to obtain a time course to characterize the concentration of tracer in arterial blood. IDIF approaches may also require at least one physical blood sample to scale the image-derived AIF curve, and may include tracer uptake information both before and after tracer metabolism. Alternatively, PET signals from multiple regions (not necessarily arterial blood pools) may be leveraged to recover AIF and thereby kinetic parameters, via simultaneous estimation of the input function [14].

Population-based input functions (PBIF) [15]-[20] are obtained by estimating a patient’s AIF by scaling an overall population arterial input pattern (obtained from historical information on the patient’s population or by averaging fits from the overall cohort) based on individual subject information (depending on availability, historical cohorts have 10-30 blood samples through 60-90 minutes of data, and have a combination of patient weight, body surface area, lean body mass or cerebellar FDG activity) [21], [22]. The choice of time points at which this information is extracted is a likely contributor to variability in estimation of the signal scale. With this approach, the individual is assumed to have the same tracer injection protocol and physiological characteristics as the population, which is not always realistic.

**Fig. 1.**
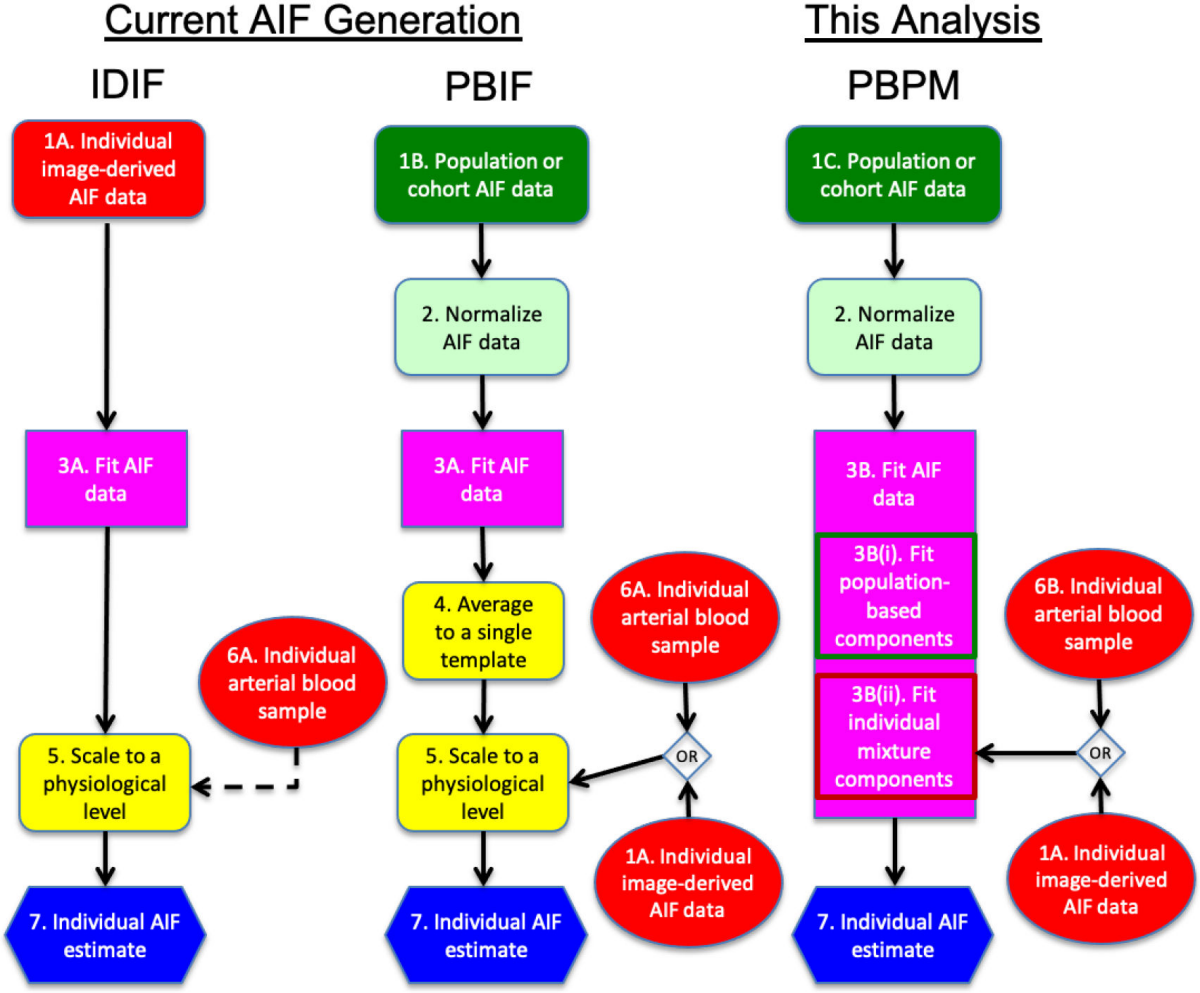
Flowchart of typical PBIF and IDIF strategies, along with the proposed PBPM methodology Red and green blocks indicate use of individual subject and population data respectively. Step 3 of each method is an AIF model fitting step. Blood sampling for step 6A is not used in some IDIF approaches (hence the dashed line); when used, fewer points are typically required compared to blood sampling used in step 6B. Using individual image-derived data (1A) at step 3B(ii) of the PBPM approach (instead of arterial sampling data) would require an extra step in order to scale the output to a physiological level, as in step 5 of an IDIF method.

Various mathematical constructions such as Feng’s model [23], tri-exponential models [24] and convoluted models [25] have been developed to provide a continuous and noise-free description of the AIF obtained from either clinically sampled (as in typical PBIF strategies) or image-derived (as in typical IDIF strategies) signals, as shown with step 3A of the flowchart in Fig. 1. The tri-exponential construction and its convolved alternative rely on a linear mixture of exponential density functions fit to sample arterial data. The tri-exponential model (TE) fits the AIF shape in two parts: a first linearly increasing piece from the time the AIF starts rising at to its peak, and a second piece modeling the AIF from its peak to the end of study using a linear combination of the three exponential curve. In comparison to Feng’s model, the TE model provides more flexibility to describe the AIF. However, neither model accounts for the length of the tracer injection and could lead to poor fit to the initial part of the AIF curve. An adaptation of the TE model was proposed [25] that includes the information of injection duration by convolving the injection profile and linear combination of three exponential curves. This convolved tri-exponential (CTE) model addresses some of the issues of the original TE model, but relies on a boxcar function of fixed duration to describe the injection profile, which used on its own may not represent the pattern of tracer arrival into plasma realistically. This particular limitation has been addressed in some works [26]-[29], by using a convolution of a double Butterworth function with the TE function [28], or of a square wave with a Gamma function [29]. Final individual AIF estimates are obtained by scaling the estimated AIF pattern to the individual physiological level using either image-derived blood level estimates from a reference region (after partial volume correction) or blood sampling data. (Some alternative scaling methods exist that use routine patient information instead; this is discussed further below.)

An alternative, compartment model based technique was developed by our group to estimate the AIF from a whole-body tracer circulation model (BCM) [29], [30]. In this representation, the fate of each tracer atom is represented by an eight-state Markov chain process discrete by heart beats, where each state represents one important location in the blood circulation system. To use the BCM, theoretically, time course of the tracer concentration from one of the eight states is required. Since the left ventricle is one of those states represented in the stochastic process, the AIF signal can be approximated by the tracer concentration in the left ventricle observed from PET imaging over time. Otherwise clinically sampled arterial data can be used for evaluation of one of these Markov states. Although high dimensionality of the parameters involved in the model constitutes a computational challenge, this physiologically meaningful representation can yield an accurate AIF estimation.

### C. Contribution

Here we consider a modified PBIF-type approach by adjusting a population AIF profile to individual characteristics extracted from either arterial sampling or image-derived data, to achieve reliable patient-adaptive AIF estimation throughout the whole time course. In a sense the proposed population-based projection model (PBPM) combines population profiling (as in a PBIF approach) with individual arterial input data modeling (as in an IDIF approach). As such, it differs from both these strategies, in a number of ways. Unlike IDIF strategies, the PBPM leverages population information, and does not depend on the availability of imaging data (although it can also be applied using such data). Unlike PBIF strategies, it does not average population components into a single individual template pattern, but instead produces a different pattern for each individual, by fitting a linear mixture of population profile components to subject-specific arterial sampling data. Fig. 1 describes the overall process and illustrates how it differs from typical PBIF and IDIF processes. To produce individual fits, the PBPM mixture components are fitted to population data, and a combination of these fits is adapted to each subject, unlike with TE-type methods. This approach could also be adapted for use in step 3B in the flowchart, but no such report was found in the literature. The proposed PBPM allows the use of functions other than exponentials, should a particular tracer require it, and can operate with a different number of components depending on the PET tracer used. This number can be automatically selected; two or three such population components are typically sufficient for individual AIF estimation. The proposed PBPM also provides more flexibility with respect to temporal characteristics of the data. It allows for injection duration to be included in the modeling, which makes it applicable to PET studies with different injection durations. Unlike TE-type constructions, it also does not require specification of two key time points (signal start and peak time), and can adapt to different peak times thanks to its parametrization. A model fitting procedure for the PBPM is also proposed hereafter. A penalized positive constrained least squares method is developed and implemented in R [31] for estimation of individual mixing weights. Statistical regularization is built in to adapt relative weight placed on the individual and population information according to the noise level in a given dataset, to facilitate patient-adaptive fitting of cohort characteristics.

This paper focuses on describing aspects related to the PBPM (step 3B in the flowchart), and benchmarking this model against reference methods typically used in step 3A of PBIF and IDIF frameworks. Section II develops the proposed model, and provides details on the methodological developments for fitting the model to clinically sampled arterial data, including regularization and cross-validation for selection of the number of components. Section III illustrates model selection and evaluation, convergence of the procedure and other aspects related to its construction, using both simulated and clinical data comprising of three separate arterial sampling datasets of respectively FDG-, H_2_O-, and FLT-PET imaging studies. Results from these numerical analyses include validation of an open source implementation of the software in R [31] that we made available [32]. Section IV presents a comparative analysis against another three methods of reference, namely the TE, CTE and BCM models. Results on clinical data demonstrate the gain derived by training the proposed AIF representation on population data, as well as its impact on kinetic analysis is evaluated via resampling of uptake templates in one- and two-tissue compartment models.

## II. Methods

This section presents the modeling strategy used for patient-specific AIF representation based on PET tracer concentration profiling, including aspects of model fitting and calibration.

### A. AIF model specifications

Let the travel time *T* of a specific atom at a specific arterial sampling site be modeled as the sum of the time (*T*_*ir*_) for the atom to initially progress from the injection site to the right ventricle (RV) of the heart, and the time (*T*_*c*_) it spends in circulation, before being sampled at the site:

$$T=T_{ir}+T_{c}$$

*T*_*ir*_ is modeled as a realization from a Gamma distribution (*G*) with shape and rate parameters *α* and *β*, respectively. The circulation time is modeled as a shifted excess circulation time (*ξ*) with the value of the shift (Δ) being specific to the location of sampled blood-site within the circulatory system

$$T_{c}=\Delta +\xi$$

If the probability density (*f*) of *ξ* is known, it is possible to combine this distribution with the injected dose profile in the right ventricle to evaluate the tracer concentration at any particular time. Under the conventional assumptions of time invariance and linearity of tracer kinetics, a bolus injection at time *t* = 0 will produce a temporal concentration of tracer atoms in the RV proportional to the distribution *G*. A more complex temporal injection profile, *e.g.* specified by an indicator function *I*(·), would yield an RV concentration profile *C*_*RV*_ characterized by an accumulation over the whole injection time, such that

$$C_{RV}(t)=G⊗I(t)=\int_{0}^{t}G(t-s)I(s)ds$$

The evolution of this profile to the concentration profile at the blood site of interest, *C*_*P*_, is governed by the circulation time distribution. This is given by a shifted convolution with the excess circulation travel time density

$$C_{P}(t)=f⊗C_{RV}(\cdot -\Delta )(t)=\int_{0}^{t}f(t-s)G⊗I(s-\Delta )ds$$

We approximate the distribution *f* of excess circulation travel time *ξ* by a linear mixture of *J* components to obtain an estimate

$$\hat{f}(\xi )=\pi_{1}e_{1}(\xi )+\pi_{2}e_{2}(\xi )+\cdots +\pi_{J}e_{J}(\xi )$$

where the *π*_1_, *π*_2_,…, *π*_*J*_ are mixing fractions of the component densities *e*_1_, *e*_2_,…, *e*_*J*_, with constraint *π*_*j*_ > 0, ∀*j* = 1,…, *J*. With this representation, an estimate of the concentration profile becomes

(1) $${\hat{C}}_{P}^{J}(t)=\sum_{j=1}^{J}\pi_{j}\int_{0}^{t}e_{j}(t-s)G⊗I(s-\Delta )ds$$

In this expression, the convolution (⊗) of the square wave *I*() with the Gamma distribution *G* describes a population injection profile, adjusted to individual subjects by a patient-specific delay Δ. In this approach the population component densities *e*_1_, *e*_2_,…, *e*_*J*_ are assumed to be standard exponentials with characteristic rates *ϕ*_1_ > *ϕ*_2_ > ⋯ > *ϕ*_*J*_ > 0. While many other possibilities could be considered (*e.g.* Gamma, Weibull, etc.), a mixture of exponential distributions makes for a simple and flexible model. We presume the mixing components are representative of the entire circulatory system, and functional components *e*_1_, *e*_2_,…, *e*_*J*_ and *G* are held fixed across subjects in the population of study.

Ambiguity may arise in the estimation of the linear coefficients ${\hat{\pi }}_{1}$ and ${\hat{\pi }}_{2}$, in cases where the corresponding distribution ranges overlap. To remove this ambiguity, a constraint *π*_1_ > *π*_2_ can be imposed when estimating these linear parameters. This constraint ensures that at early time points, the first component contributes mostly to the peak of AIF representation. A similar constraint may be applied to further mixing coefficients, although in our experience on the data of Section IV, it was not necessary as there was no practical ambiguity between *π*_2_ and *π*_3_ in a three-component mixture applied to dynamic FDG-PET data.

### B. Model fitting

Suppose blood time course data from a training population of *n* subjects

$$\left\{(t_{ik},z_{ik},w_{ik}),\;i=1,2,\ldots ,m_{k},\;k=1,2,\ldots ,n\right\}$$

are available, where *z*_*ik*_ is the concentration of the *i*th measurement of the *k*th subject, measured at time *t*_*ik*_ with measurement reliability *w*_*ik*_ and *m*_*k*_ is the number of time points used in clinical sampling for the *k*th subject. Each time course *z*_*ik*_ is modeled for *i* = 1, 2, … , *m*_*k*_, *k* = 1, 2, … , *n* as

$$z_{ik}={\hat{C}}_{P}(t_{ik}|\Delta_{k},\pi_{k},\theta )+w_{ik}^{-1∕2}\varepsilon_{ik},$$

where ${\hat{C}}_{P}(t|\Delta_{k},\pi_{k},\theta )$ is defined in the previous subsection with the subject-dependent linear parameter vector *π*_*k*_ = *c*(*π*_1*k*_, *π*_2*k*_, … , *π*_*Jk*_), and subject-independent non-linear parameter vector *θ* = *c*(*α*, *β*, *ϕ*_1_, … , *ϕ*_*J*_). The *ε*_*ik*_’s are assumed to be i.i.d realizations of Gaussian random variables modeling additive noise. We propose to estimate *θ* by minimizing the objective function defined by

$$l(\theta )=\sum_{k=1}^{n}\underset{\Delta_{k},\pi_{k}}{\text{min}}\sum_{i=1}^{m_{k}}w_{ik}(z_{ik}-{\hat{C}}_{P}(t_{ik}|\Delta_{k},\pi_{k},\theta ){)}^{2}$$

An algorithm for this optimization is implemented in R [31]. Minimization of *l*(*θ*) requires two-step estimation of *π*_*k*_ and Δ_*k*_, which is described in the following two subsections, respectively.

#### 1) *Estimation of* *π*_k_ *given* Δ_k_ *and θ:*

The linear mixture is fitted by minimizing, for *k* = 1 , … , *n*, criterion

$$l_{1}(\pi_{k}|\Delta_{k},\theta )=\sum_{i=1}^{m_{k}}w_{ik}(z_{ik}-{\hat{C}}_{P}(t_{ik}|\Delta_{k},\pi_{k},\theta ){)}^{2}\;\;=\sum_{i=1}^{m_{k}}w_{ik}{\left(z_{ik}-\sum_{j=1}^{J}\pi_{jk}A_{ijk}\right)}^{2}$$

with respect to *π*_*k*_ given Δ_*k*_ and *θ*, where the *j*th component of the linear mixture for the *k*th patient, hereafter denoted by *A*_*ijk*_ for convenience, can be calculated as

$$A_{ijk}=A_{j}(t_{ik}|\Delta_{k},\theta )=\int_{0}^{t_{ik}}e_{j}(t_{ik}-s)G⊗I(s-\Delta_{k})ds$$

for *i* = 1, … , *m_k_*, *j* = 1, … , *J*, where the mixing parameters $\{\pi_{jk}{\}}_{j=1}^{J}$ are constrained positive. An R implementation of the Goldfarb-Idnami method [33] is employed to solve this positivity-constrained least-squares problem. We denote the resulting minimizer of *l*_1_(*π*_*k*_∣Δ_*k*_, *θ*) as ${\hat{\pi }}_{k}=\hat{\pi }(\Delta_{k},\theta )$.

#### 2) *Estimation of delay* Δ_k_ *given θ:*

Inserting ${\hat{\pi }}_{k}(\Delta_{k},\theta )$ in the objective function *l*_1_, we have the reduced objective function

$$l_{2}(\Delta_{k}|\theta )=l_{1}({\hat{\pi }}_{k}(\Delta_{k},\theta )|\theta )$$

Given population characteristics *θ*, a grid search may be carried out to minimize *l*_2_(Δ_*k*_∣*θ*) with respect to Δ_*k*_. The measured data may not be coincident with the timing of the injection, and a parameter Δ_*k*_, positive or negative, is introduced to account for this. In this study, 21 evenly distributed grid points in the range of [−60 s, +60 s] were used. The resulting minimizer of *l*_2_ is denoted as ${\hat{\Delta }}_{k}$.

#### 3) Estimation of population parameter θ:

Finally, the population-specific parameters *θ* = (*α, β, ϕ*_1_, … , *ϕ*_*J*_) are estimated by minimizing

$$l(\theta )=\sum_{k=1}^{n}\sum_{i=1}^{m_{k}}w_{ik}(z_{ik}-{\hat{C}}_{P}(t_{ik}|{\hat{\Delta }}_{k},{\hat{\pi }}_{k},\theta ){)}^{2}$$

Initial values for $\theta^{0}\;=\;(\alpha^{0},\beta^{0},\phi_{1}^{0},\ldots ,\phi_{J}^{0})$ may be selected based on experience from previous experiments and some physiologic reasoning about the average travel time of each type of tracer in the blood circulation system. Lower and upper bounds $\theta^{L}\;=\;(\alpha^{L},\beta^{L},\phi_{1}^{L},\ldots ,\phi_{J}^{L})$ and $\theta^{H}\;=\;(\alpha^{H},\beta^{H},\phi_{1}^{H},\ldots ,\phi_{J}^{H})$, respectively, can be set based on experience from the population of patients receiving the same tracer injection, and physiologic reasoning. Since the scales of the parameters differ considerably, and also to avoid the complexity of constrained minimization, the following parameter transformation may be performed. Let $p=\frac{\theta -\theta^{L}}{\theta^{H}-\theta^{L}}$ be transformed via a logit transform into

$$\varsigma =\log\;\frac{p}{1-p}$$

Inversely,

$$\theta =\theta^{L}+(\theta^{H}-\theta^{L})\frac{e^{\varsigma }}{1+e^{\varsigma }}$$

Thus, the constrained minimization with respect to *θ* can be performed as unconstrained minimization of *l*(*ς*) with respect to *ς*. A Gauss-Newton type method was implemented in R to optimize *l*(*ς*).

#### 4) Fitting individual curves:

The population parameter estimates $\hat{\theta }={\text{arg min}}_{\theta }\;l(\theta )$ defined in Step (II-B.3) can be used instead of being re-evaluated when fitting time activity curves for “new” patients (i.e. patients outside of the training population of *n* studies used to obtain $\hat{\theta }$), thus reducing the number of parameters required to obtain an individual fit. In such cases, simultaneous estimation of *π*_*k*_ and Δ_*k*_ can be achieved by grid search over *G* values $\left\{{\hat{\Delta }}_{k}^{1},\ldots ,{\hat{\Delta }}_{k}^{G}\right\}$, corresponding estimates $\left\{{\hat{\pi }}_{k}^{1},\ldots ,{\hat{\pi }}_{k}^{G}\right\}$ being obtained instantaneously via linear regression.

#### 5) Model regularization:

In cases of low signal-to-noise ratio^1^, regularization of the patient-specific mixing weights *π* can be applied to control variability of these estimates. Once the number and parameters of the population components have been determined, a penalized nonlinear least squares approach using the historical linear parameters as a Bayesian penalty for individual fit [34] is used for this approach. Regularization on *π* = *π*_*k*_ parameters for the *k*th patient is achieved using the following individual objective function:

(2) $$f_{\lambda }(\pi |\Delta ,\theta )=W(z^{T}-\pi^{T}A_{\Delta }^{\;\;T}{)}^{2}\;\;+\lambda (\pi^{T}-\pi_{0}^{\;\;T})\Omega^{-1}(\pi^{T}-\pi_{0}^{\;\;T}{)}^{T}\;\;=-[(A_{\Delta }^{\;\;T}(\sqrt{W}z){)}^{T}+\lambda \pi_{0}^{\;\;T}\Omega^{-1}]\pi \;\;+\pi^{T}(A_{\Delta }^{\;\;T}WA_{\Delta }+\lambda \Omega^{-1})\pi$$

where $z=\{z_{ik}{\}}_{i=1}^{m_{k}}$, *A*_Δ_ = [*A_ijk_*]_*i,j*_ is a matrix of dimension *m*_*k*_ × *J*, and *W* = *W*_*k*_ are defined in Section II-B.1. All components in the vectorized mixing weights *π* are constrained to be nonnegative, and *π*_0_ and Ω respectively denote the mean and covariance matrix of linear parameters obtained from population-based estimation. regularization parameter λ controls the trade-off between individual and population data contributions, larger values of *λ* constraining individual estimates to be closer to the population profile *π*_0_, which suits higher-noise scenarios. The optimization is a quadratic problem for which we used the Goldfarb-Idnami method [33].

Parameter *λ* is set by minimizing the generalized cross-validation (GCV) score which is defined as the mean squared error adjusted by the effective degree of freedom:

$$GCV(\lambda )=\frac{\frac{1}{m}\sum_{i=1}^{m}(z_{ik}-{\hat{z}}_{ik}(\lambda ){)}^{2}}{{\left(\frac{1}{m}\text{Trace}(I-A_{\Delta }(A_{\Delta }^{\;\;T}A_{\Delta }+m\lambda I{)}^{-1}A_{\Delta }^{T})\right)}^{2}},$$

where ${\hat{z}}_{ik}(\lambda )$ is ${\hat{C}}_{P}(t_{ik}|\Delta_{k},\pi_{k},\theta )$ based on minimization of the objective function defined in Eqn. (2), for a given λ.

### C. Model selection

Similarly to compartmental analysis of dynamic PET data, the number of components needed in the linear mixture defined in Eqn. (1) may depend on the tracer used in the studies. More components may be included if the tracer molecules can participate in metabolic activity. For example, in water studies, tracers do not participate in any metabolic activity, and a one-tissue compartmental model is normally used for water dynamic studies. Models using more than one compartment are applied to studies involving other tracers for more complicated circulatory or metabolic systems [4].

Here a leave-one-out approach is developed for selection of the number of components to be used in the proposed linear mixture defined in Eqn. (1) so as to minimize model complexity defined as

(3) $$Err(J)=\sum_{k=1}^{n}\underset{\Delta_{k},\pi_{k}}{\text{min}}\sum_{i=1}^{m_{k}}\frac{w_{ik}}{m_{k}}(z_{ik}-C_{P}^{J}(t_{ik},\Delta_{k},\pi_{k},{\hat{\theta }}_{k}){)}^{2},$$

where *J* = 2, 3 … is the number of components, $C_{P}^{J}(\cdot )$ defines the predicted AIF based on PBPM with *J* components given linear and non-linear parameters, and

$${\hat{\theta }}_{k}=\text{arg}\underset{\theta_{k}}{\text{min}}\sum_{j\neq k}^{n}\underset{\Delta_{k},\pi_{k}}{\text{min}}\sum_{i=1}^{m_{k}}\frac{w_{i,k}}{m_{k}}{\left(z_{i,k}-C_{P}^{J}(t_{i,k},\Delta_{k},\pi_{k},\theta_{k})\right)}^{2}$$

A cross-validated error (Err^) may not necessarily be the minimum model complexity *Err* for the chosen *J*, but should be reasonably small for all values *Err*(*J*) in the range of *J*.

### D. Scale factor estimation

An overarching objective of AIF estimation is to design methods that can rely on image-derived input data instead of invasive sampling. In this context, scaling of PBPM estimates of the AIF obtained from image-derived arterial signals is required to obtain final parametric imaging estimates. This evaluation typically requires at least one blood sample point (from either arterial or venous blood) [15], [35], [36], or an adequately calibrated image-derived measurement from reference tissue (e.g. cerebral tissue) [22], [37], which is not always feasible. Routine individual subject information may alternatively be used to this end. In [37] the authors use FDG uptake measured in cerebellum ROI from a single scan acquisition to normalize a PBIF estimate. In [38] the authors show that body surface area can be used as an alternative to blood sampling in PBIF normalization in brain ^11^C-TMSX PET studies. In [39], the authors use injection dose and weight information to scale PBIF estimates in ^11^C-DPA-713 PET studies. Here we propose a regression model for the scale factor that does not require arterial blood sampling or a large blood-pool in the field of view. This model uses injection dose *X*_*ID*_, blood volume *X_BV_* (estimated from non-invasive patient information on body weight, height and total blood volume [40]) and tail height of the AIF curve (${\hat{\pi }}_{3}$, the linear coefficient for the third component in the PBPM) as independent variables:

(4) $$log(S)=\beta_{0}+\beta_{1}X_{ID}+\beta_{2}X_{BV}+\beta_{3}{\hat{\pi }}_{3}+\text{noise}$$

## III. Evaluation

This section introduces the three clinical datasets used for evaluation of the proposed PBPM model. Model selection, evaluation of the impact of training population size on model fitting, and scaling of the final AIF estimates are covered in these analyses. Complementary simulations based on the sampling data also illustrate convergence of the procedure at varying noise levels.

### A. Data

**Fig. 2.**
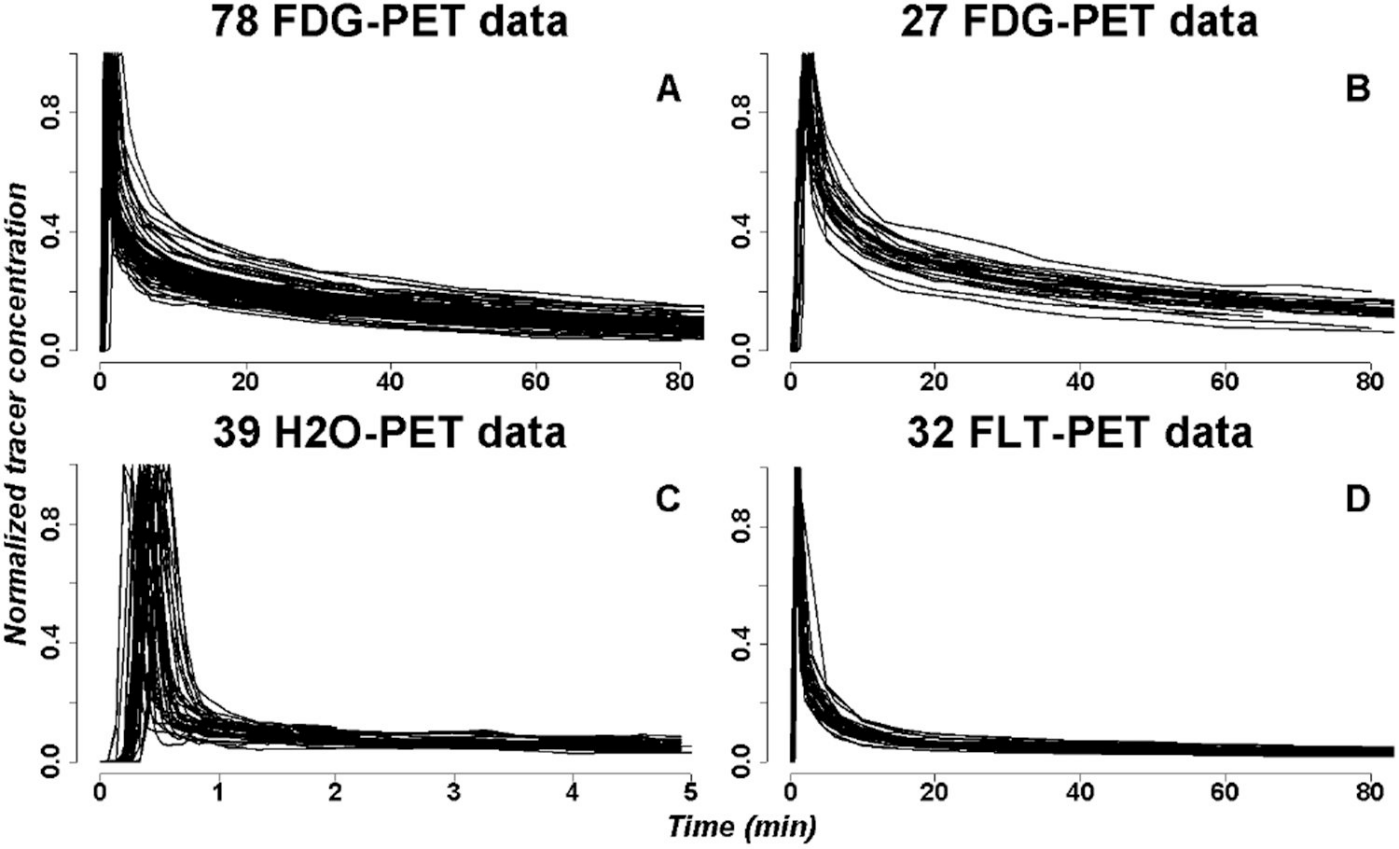
Normalised kinetic PET time activity curves acquired at the University of Washington [9], [29], [41], [43], [44]: (A) 78 FDG-PET curves with 1-minute injection duration, (B) 27 FDG-PET curves with 2-minute injection duration; (C) 39 H_2_O-PET curves with bolus injection; (D) 32 FLT-PET curves with 1-minute injection.

The proposed model was applied to three separate datasets, comprising blood sampling data obtained during the acquisition of respectively 105 FDG-PET studies, 39 H_2_O-PET studies, and 32 FLT-PET studies, all acquired at the University of Washington [9], [29], [41]-[44]. The data were from both healthy subjects and patients with cancer conditions. All FLT patients had cancer (brain, breast, lung, sarcoma). Of the 105 FDG blood curves used in the analysis, 12 were from normal human subjects with the rest from patients with cancer, including brain, lung, liver and colon cancer patients. Eighteen of the 39 water curves came from normal subjects. Durations of FDG injections were either 1 or 2 minutes (58 and 33 cases respectively), bolus injection for H_2_O was assumed to be 5 seconds, and duration of FLT injection was 1 minute. The datasets are summarised in Table I. The data included sample AIFs of the subjects along with gender, weight, height and injection dose. Median sampling durations and numbers of sampling time points per tracer are also summarised in that table. The most common sampling frame for FDG was as follows (min): 0, 0.25, 0.5, 0.75, 1, 1.5, 2, 2.5, 3, 4, 5, 6, 7, 10, 13, 15, 20, 25, 30, 35, 40, 50, 60, 70, 80, 90. For H_2_O, it was 0, 0.067, 0.13, 0.20, 0.27, 0.33, 0.40, 0.47, 0.53, 0.60, 0.67, 0.73, 0.80, 0.87, 0.93, 1.08, 1.27, 1.42, 1.58, 1.75, 1.92, 2.25, 2.58, 2.92, 3.25, 3.58, 3.92, 4.25, 4.58, 4.92. For FLT, it was 0, 0.25, 0.5, 0.75, 1.00, 1.25, 1.5, 1.75, 2, 2.5, 3, 4, 5, 6, 7, 8, 10, 15, 20, 30, 40, 50, 60, 90, 110. All water studies were manual bolus injections. Only the initial 7 FDG studies were manual injections over 1 min. The other FDG studies had either a 1 min or 2 min programmed infusion using a syringe pump. All FLT injections were a 1 min injection from an infusion pump. A saline flush was used after every injection. Ten studies from each of these 3 sets were used for PBPM fitting of the population components *θ*. The rest of the data were used to obtain individual fits from each of the models used in the following comparative analyses. The normalized AIFs are presented in Fig. 2. Each curve typically includes three parts: a plateau of 0 concentration at the begin, followed by a rapid increase after injection, and an exponential decay. No imaging data were used in the following analyses.

### B. Model selection

**Fig. 3.**
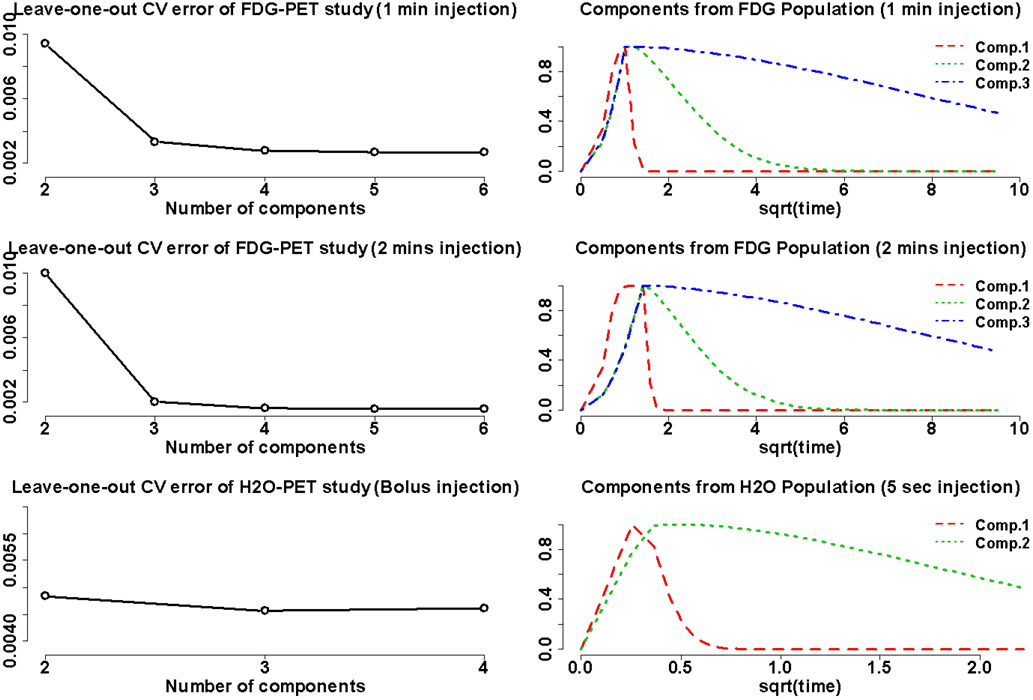
Left: model selection for FDG (top for one-minute injections; centre for two-minute injections) and H_**2**_O (bottom) studies on the basis of the leave-one-out cross-validated error defined in Section III.C. Right: corresponding basis component fits.

The clinical datasets were used to estimate the population-based parameters in the proposed PBPM model for varying numbers of components. Figure 3 depicts the cross-validated error Err^ defined from *Err*(*J*), for the FDG- and H_2_O-PET cohorts respectively, after carrying out the model selection procedure described in Section II-C. For the FDG set an elbow was clearly identified at 3 components, indicating a 3-component mixture to be optimal in the sense of loss *Err*(*J*). Three exponentials with different rates convolved with a Gamma distribution were thus estimated from the population data to approximate the travel time distribution of the FDG and FLT tracers, yielding a reasonable representation in PBPM (which aligns with the traditional two-tissue compartment modeling of FDG molecules in the blood circulation system). For the H_2_O set, there was no clear elbow and a 2-component mixture seemed the optimal choice according to this selection method. Since, unlike FDG, H_2_O is not involved in the metabolic process, it seems reasonable that the number of components used to describe H_2_O AIF might be one less than FDG. Note that this model selection rule is only indicative, and using an over-specified mixture (i.e. using more components) may also mechanically impact the bias-variance tradeoff achieved by the model.

### C. Model fitting

Model fitting was analysed on both clinical data (Section III-C.1) and simulated data created using the clinical data as templates (Section III-C.2), to illustrate the procedure and demonstrate its convergence and reliability at varying noise levels.

#### 1) Model fitting:

**Fig. 4.**
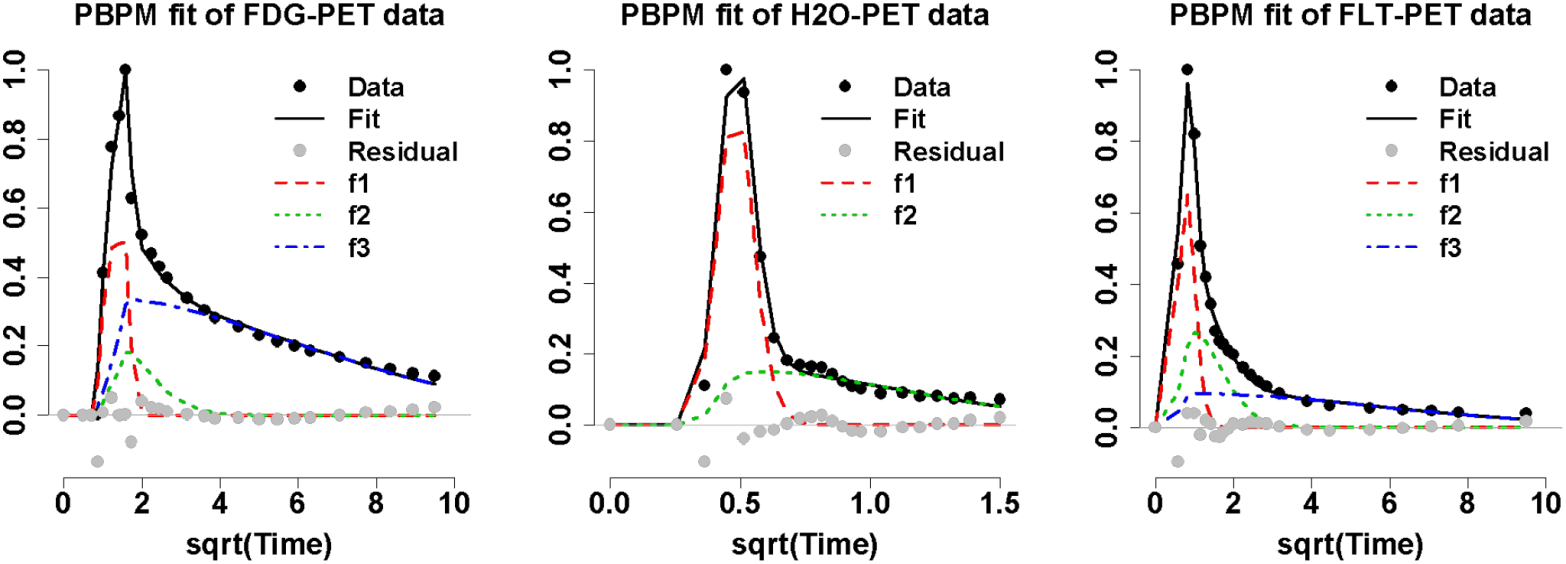
Examples of individual PBPM fits to FDG (left), H_2_O (middle) and FLT (right) arterial data, where the population-fitting components ***f**_**j**_* shown in each plot, defined as the convolutions between the exponential components ***e**_**j**_* with the population Gamma profile ***G*** in Eqn. (1), are scaled by individual mixing weights **π**_***j***_ (with ***j*** = **1, 2, 3** for FDG and FLT data and ***j*** = **1, 2** for H_2_O data).

Figure 4 shows examples of AIF fits obtained from PBPM for FDG-, H_2_O-, and FLT-PET clinical studies based on the above model selection. These examples of individual PBPM fits illustrate how the third component in the FDG and FLT PBPM fits, and the second component in the H_2_O PBPM fit, could adequately represent the sample AIF tail patterns.

#### 2) Model validation and assessment:

**Fig. 5.**
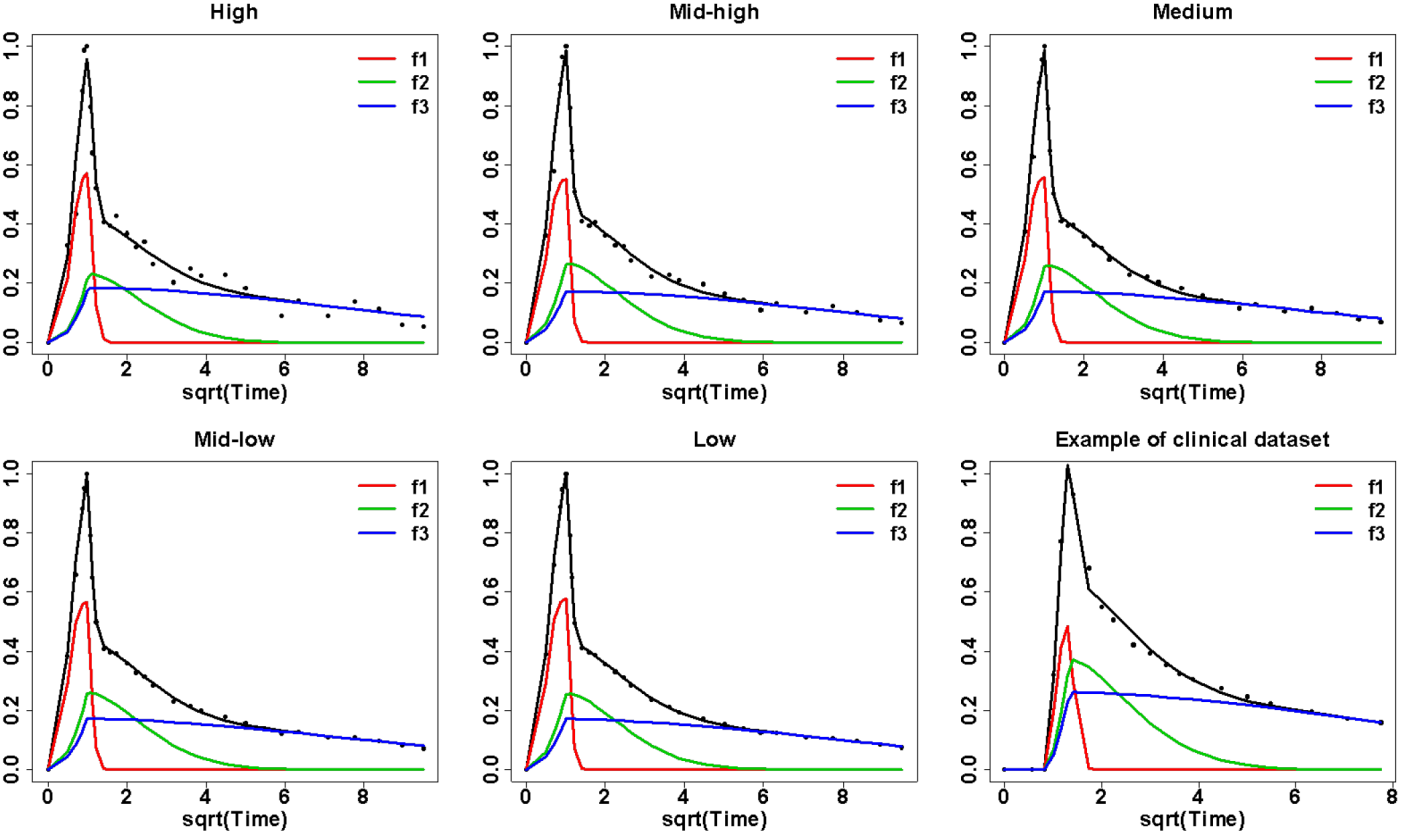
Example of PBPM fit on a simulated curve for each noise level.

A set of complementary simulation-based analyses were conducted to validate the model and its implementation. A first analysis was carried out on AIF templates generated by adding random noise to the PBPM model curve, to validate the implementation and evaluate model performance at varying noise levels. Details on the simulation settings used for these analyses are provided in Appendix A. Figure 5 presents one example simulated curve and its fit for each noise level.

**Fig. 6.**
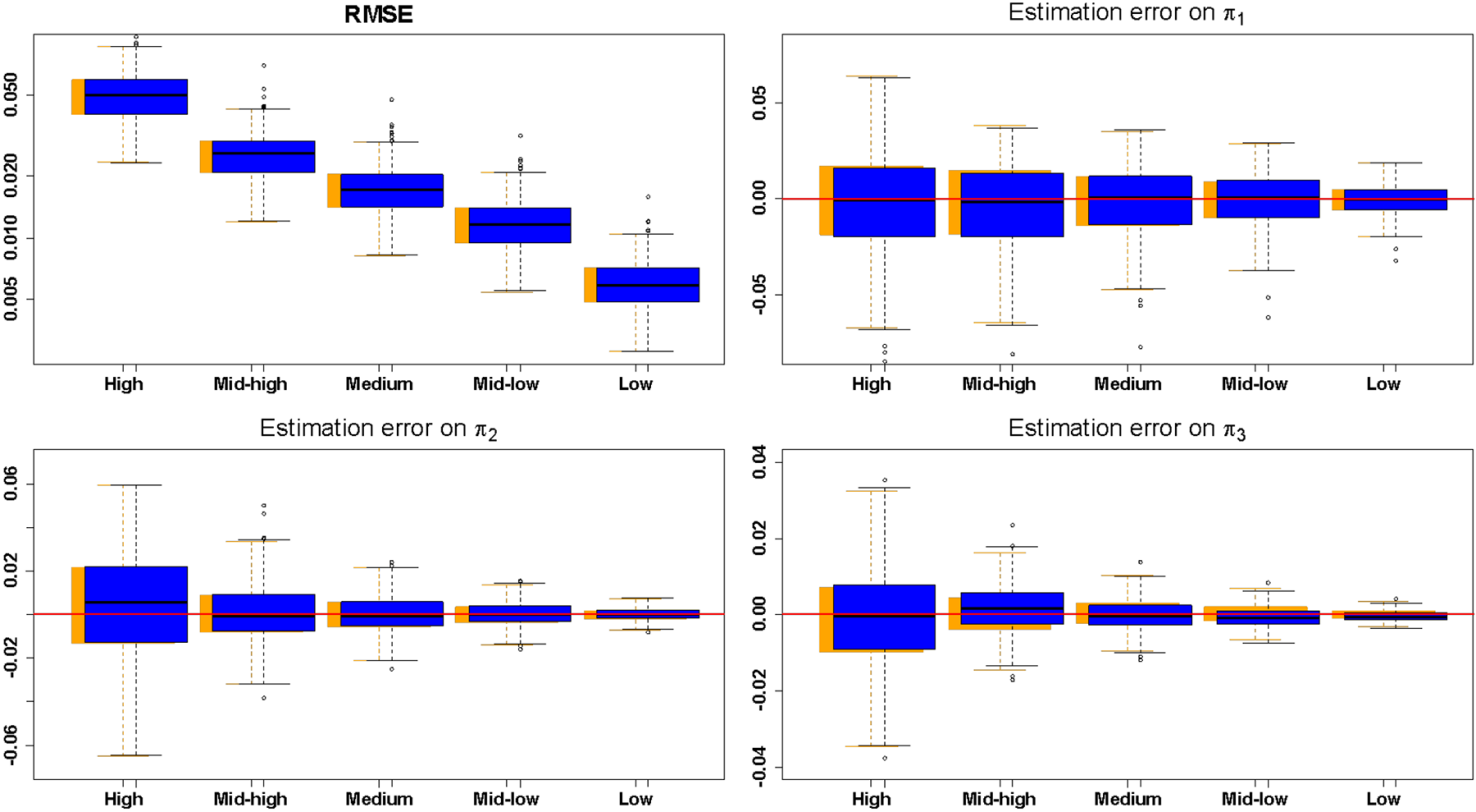
Top-left: root mean square error (RMSE) distributions for the 400 AIF fits obtained from PBPM at each noise level. Top-right and bottom: distributions of estimation errors $\pi_{i}-{\hat{\pi }}_{i}$, ***i*** = **1, 2, 3**. Blue boxplots represent the distributions of parameter estimates obtained using estimated nonlinear parameters for ***θ*** = (***α***, ***β***, ***ϕ***_**1**_, ***ϕ***_**2**_, ***ϕ***_**3**_), whilst the orange (shadow) boxplots indicate those same distributions when the true values for the nonlinear parameters were used.

Figure 6 presents the performance of PBPM on 400 simulated AIFs where medium noise level is as above when the noise pattern is set to be similar to the residuals obtained from fitting the PBPM model to the directly sampled AIFs. The RMSE distributions showed overall improvement in model fit as noise level decreases, as expected. The parameter estimation error distributions in Fig. 6 showed an increase in estimation efficiency as noise level decreases. The figure also illustrates that model performance using nonlinear parameters estimates for *θ* = (*α*, *β*, *ϕ*_1_, *ϕ*_2_, *ϕ*_3_) was comparable to that obtained when using the true population parameter values. These results demonstrate that the PBPM approach is consistent and accurate, and validate its implementation [32].

### D. Sensitivity to training population size

**Fig. 7.**
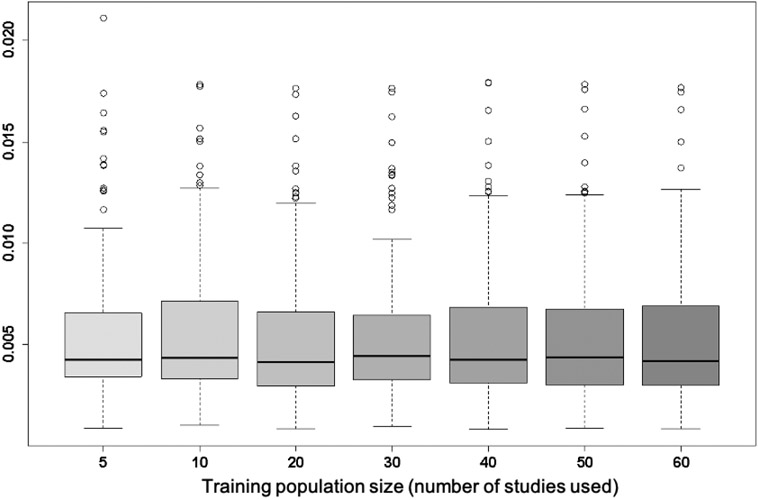
Distribution of 3-fold cross-validated AIF PBPM fit errors for varying training population sizes *n*.

As for the training population size required to fit the mixture components, behaviour of the PBPM model may vary with the PET tracer used. An experiment on the FDG cohort was carried out, using random subsets ranging from *n* = 5 to *n* = 60 studies taken from the overall set as training population studies, and monitoring cross-validated model fit errors. The results from this analysis, shown in Fig. 7, indicated that model fitting was relatively insensitive to this parameter, with comparable error distributions obtained when using population parameter estimated across the range of population sizes.

### E. Scale factor estimation

**Fig. 8.**
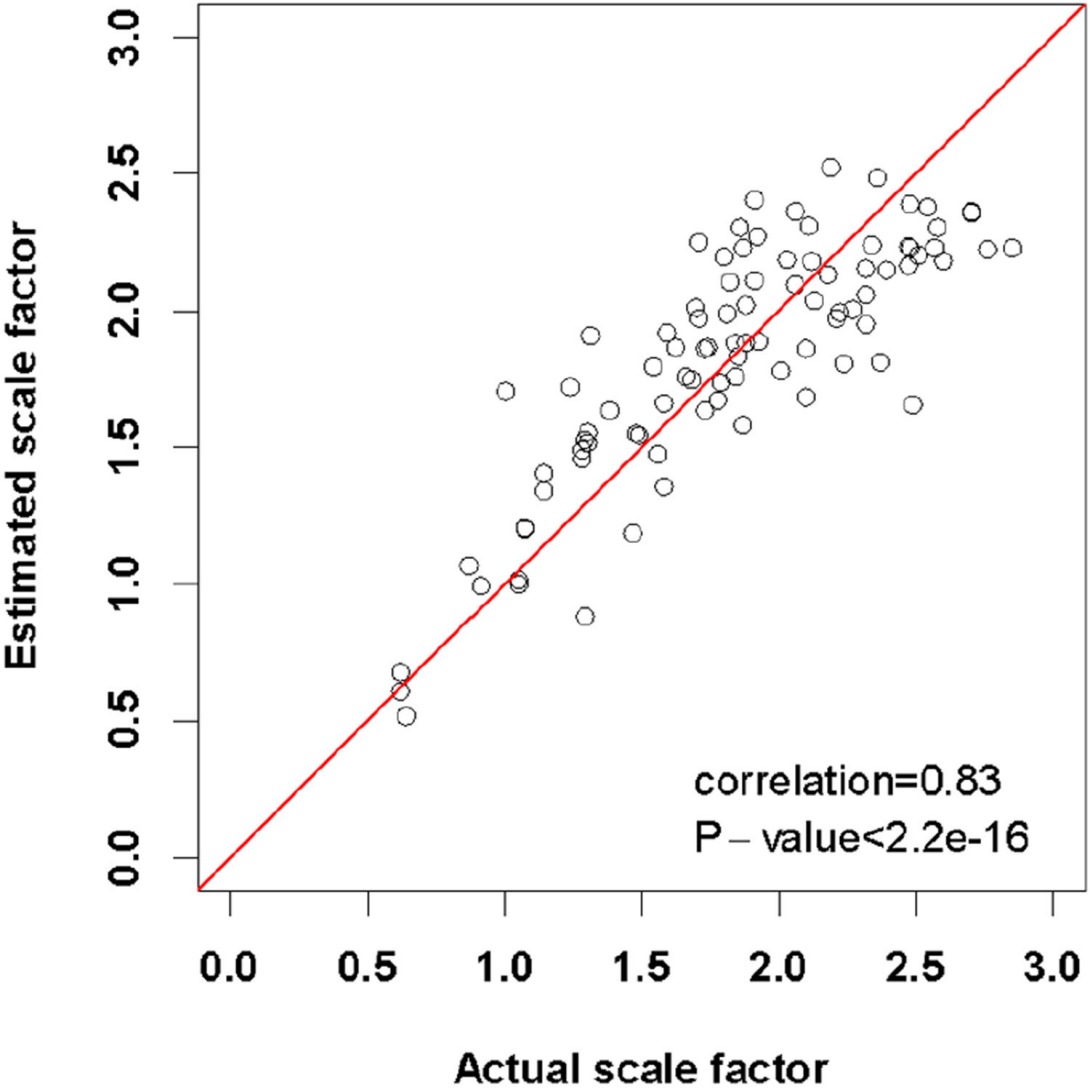
Scatterplot of estimated scale factors defined in Eqn. (4) against actual values obtained directly from arterial sampling data from 91 FDG AIF studies, showing a significant association between the two samples.

Results on 91 FDG AIF studies from the scale factor estimation procedure described in Section II-D are depicted in Fig. 8 (4 patients were removed from the set of 95 curves used in other analyses, due to missing information). They show significant alignment between estimated scale factors (Eqn. (4)) and actual scale factor values obtained directly from arterial sampling data (Pearson correlation *ρ* = 0.83, two-sided test with *p* < 1*e*^−10^ under the null hypothesis *H*_0_ : *ρ* = 0).

## IV. Results

This section provides output of comparative analyses between the proposed PBPM and reference approaches, using the three clinical datasets introduced in Section III-A. The benefit of using basis components fitted to separate population data is also evaluated hereafter.

### A. Comparative analysis

PBPM-derived AIF estimates were compared to alternatives obtained from another three models described in Section I-B, namely a whole-body blood circulation model (BCM) [29], [30], a straightforward tri-exponential model (TE) [5], [23], and a convolved tri-exponential model (CTE) [25]. For each tracer, the PBPM was fitted using a random sample of 10 arterial blood sampling curves for estimation of the population parameters *θ* = (*α*, *β*, *ϕ*_1_, …, *ϕ*_*J*_) (and not for any other step), and the rest of the data for estimation of individual AIF signals, i.e. of {(*π*_*k*_, Δ_*k*_), *k* = 1, … , *n*} (and not for any other step). In other words, the 10 randomly selected “population curves” were used as “historical data” in this framework. These were only used for the purpose of fitting the PBPM, and not the BCM, TE and CTE models. A comparative analysis between these four models was carried out in terms of the sum of mean squared errors obtained for each of the *n* scaled AIF fits $\{{\hat{z}}_{i,k}={\hat{C}}_{P}(t_{i,k}|{\hat{\pi }}_{k}){\}}_{k=1}^{n}$, defined by

$$E=\sum_{k=1}^{n}\left(\frac{\sum_{i=1}^{m_{k}}(z_{i,k}-{\hat{z}}_{i,k}{)}^{2}}{m_{k}}\right)$$

This fit error was cross-validated in the comparative analysis, using

(5) $$\hat{E}=\sum_{k=1}^{n}\left(\frac{\sum_{i=2}^{m_{k}-1}(z_{i,k}-{\hat{z}}_{i,k}^{*}{)}^{2}}{m_{k}-2}\right)$$

for comparison, where ${\hat{z}}_{i,k}^{*}\;=\;\eta^{*}(t_{i,k}|{\hat{\pi }}_{k}^{*})$ and $\eta^{*}(t_{i,k}|{\hat{\pi }}_{k}^{*})$ is the set of optimal parameter estimates for the data $\{(t_{j,k},z_{j,k}){\}}_{j=1,j\neq i}^{m}$.

**Fig. 9.**
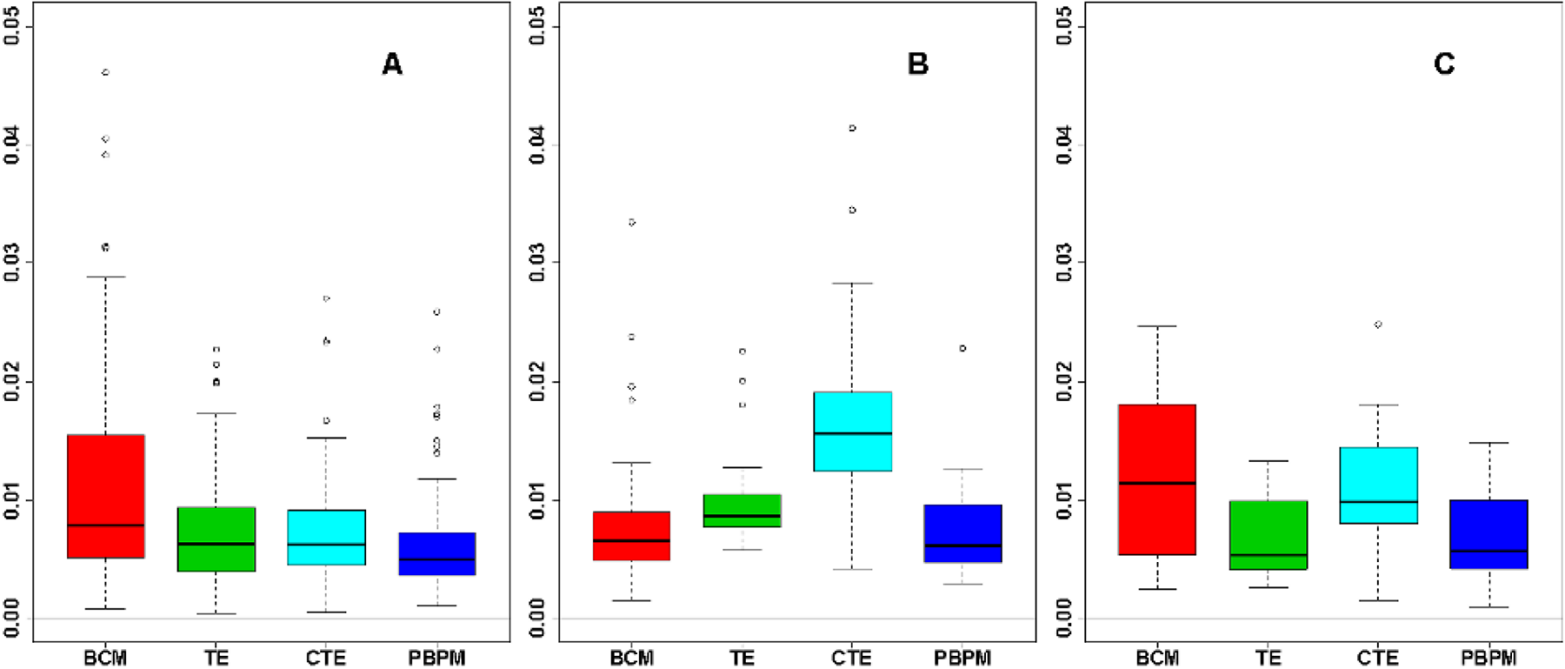
Model performance comparison for BCM, TE, CTE and PBPM on the FDG- (A), H_2_O- (B) and FLT- (C) PET studies. Distributions of cross-validated errors (Eqn. (5)) depict the relative fitting performance of the four models, and indicate that PBPM remains competitive for different tracers.

The boxplots of cross-validated error distributions of Fig. 9 (bottom) illustrate that PBPM is competitive for all three tracers. For the FDG set, the PBPM model yields comparable performance and improved accuracy over TE, and outperforms BCM both in terms of median error and standard error. For the H_2_O set, the BCM and PBPM models show greater flexibility in fitting the AIF data compared to the tri-exponential alternatives, and PBPM also yields a lower median error compared to BCM. For the FLT set, TE and PBPM perform comparably and both models outperform BCM and CTE in terms of variability and median errors. Nonparametric, one-sided (Mann-Whitney) tests under the null hypothesis of no difference in model error distribution locations (Table II) indicated a significantly lower PBPM error compared to TE and CTE for both the FDG and H_2_O sets at the 1% significance level.

### B. Gain from a population-based approach

**Fig. 10.**
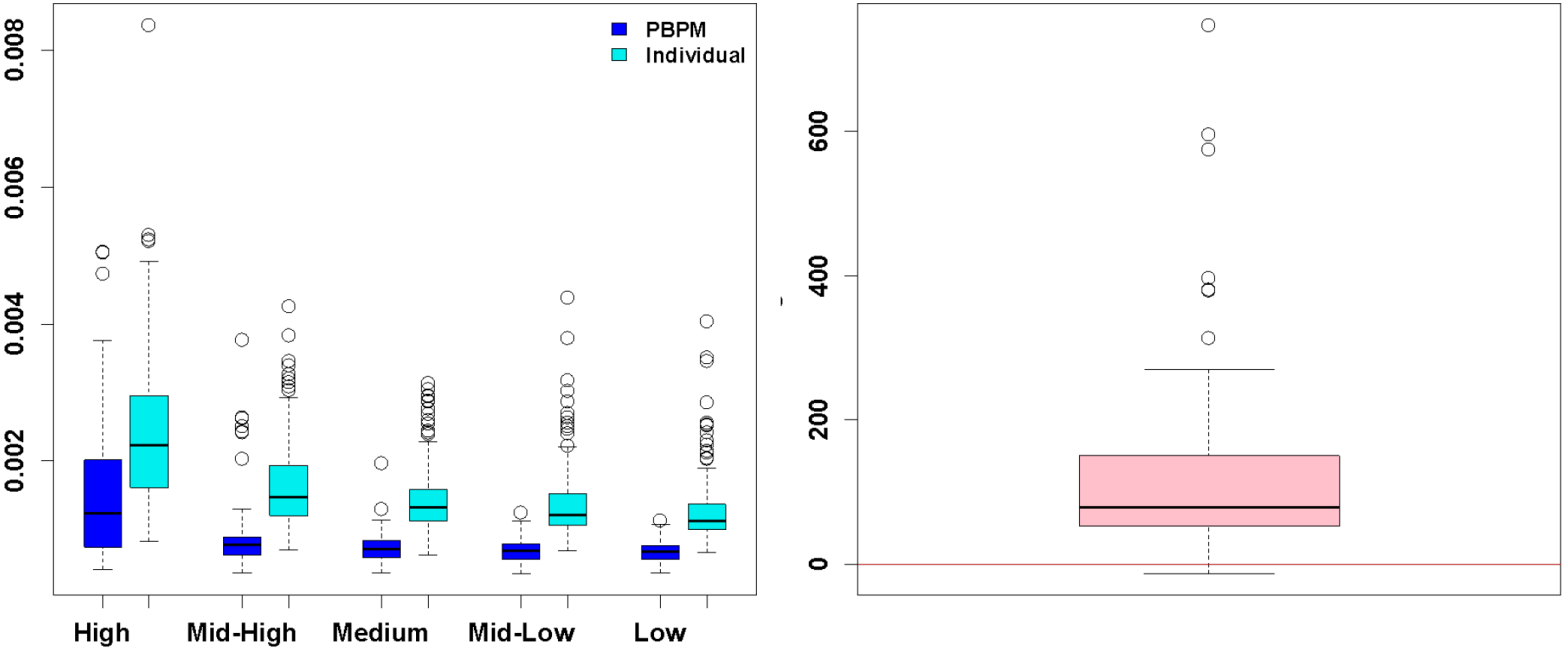
Left: distributions of PBPM and individual leave-one-out cross-validated fit errors (respectively darker and lighter coloured) at varying noise levels. Right: distribution of percentage differences between these PBPM and individual fit errors $\left(({\hat{E}}_{\mathrm{indiv}}-{\hat{E}}_{\mathrm{PBPM}})∕{\hat{E}}_{\mathrm{PBPM}}\times 100\right)$.

An initial numerical experiment was carried out on simulated data to assess the potential gain offered by employing a population-based strategy over a fully individual approach to estimate the basis components when fitting the PBPM to sampling data. The same simulation process defined by Eqn. (11) was applied to generate 210 simulated curves from the PBPM templates. A random set of 10 of these curves were used as a training population to obtain estimates $\hat{\theta }$ of the nonlinear parameters *θ*. The remaining 200 curves were fitted once following the proposed methodology (using this estimate $\hat{\theta }$ to generate PBPM fits), and independently a second time by re-estimating the nonlinear parameters *θ* individually (i.e. without using the estimate derived from the training population). We refer to the former AIF estimate as the PBPM fit, and the latter AIF estimate as the individual fit. Fit errors were calculated for each of the 200 curves and cross-validated using leave-one-out cross-validation. Figure 10 shows the distributions of cross-validated fit errors obtained from each of the PBPM and individual fits, at varying noise levels. The results show a clear gain resulting from the population-based methodology.

**Fig. 11.**
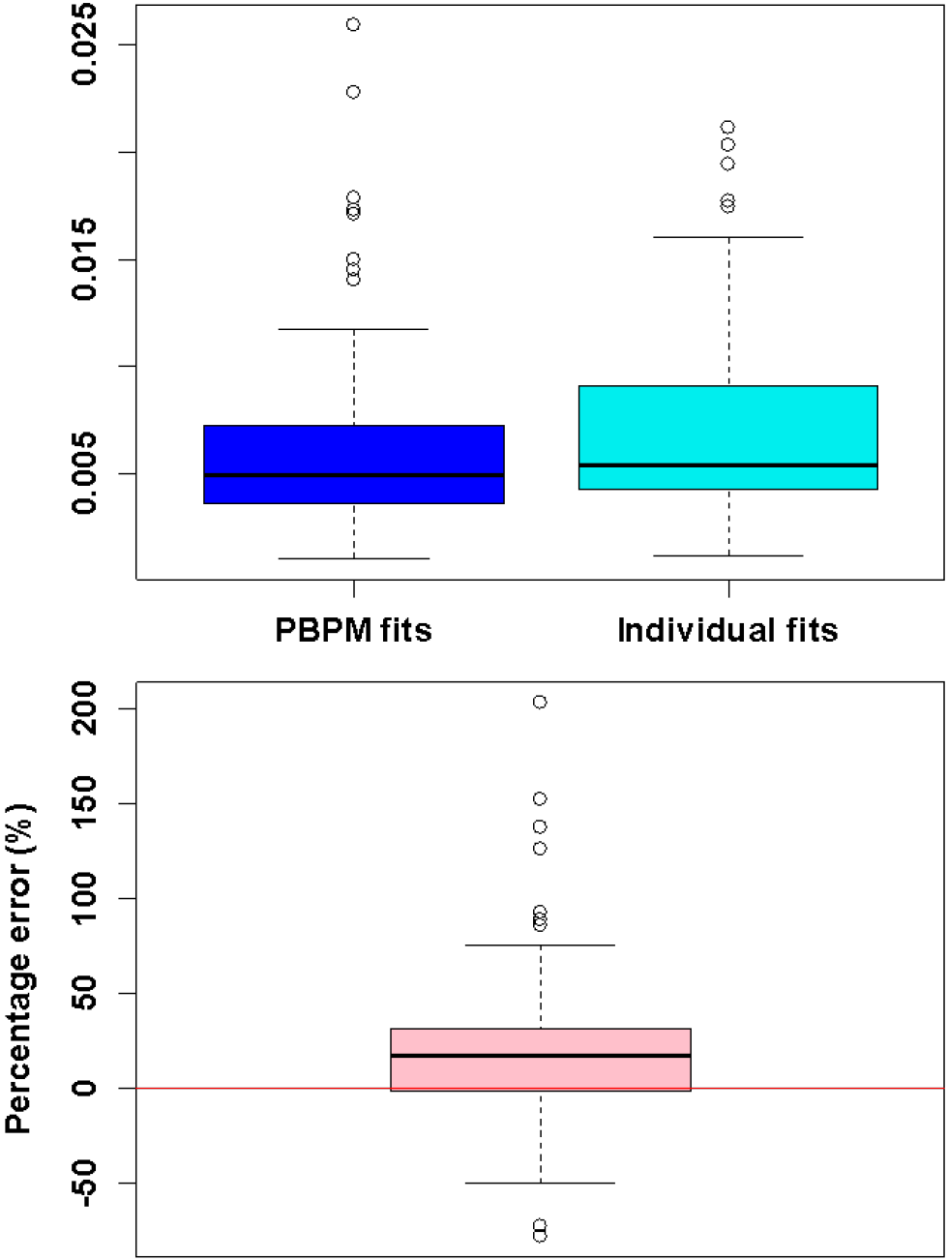
Top: comparison of cross-validated errors obtained when using a training population strategy to estimate the nonlinear parameters ***θ*** that determine the structure of the PBPM basis components (left), over a fully individual approach where the parametric vector ***θ*** is estimated directly from the same individual sampling data (right), showing a lower error tends to be achieved from a population-based strategy (one-sided paired Wilcoxon test ***p*** < **0.0001**). Bottom: direct comparison based on percent differences between these PBPM and individual fit errors $\left(({\hat{E}}_{\mathrm{indiv}}-{\hat{E}}_{\mathrm{PBPM}})∕{\hat{E}}_{\mathrm{PBPM}}\times 100\right)$.

A second analysis was carried out on the clinical data to confirm these findings. Figure 11 illustrates the gain obtained by using a population-based strategy to estimate the nonlinear parameters *θ* for the PBPM basis component profiles, over a fully individual fitting process where all model parameters (*π*, Δ, *θ*) would be estimated from the single patient sampling data. A one-sided paired Wilcoxon test, under the null hypothesis that the average cross-validated PBPM error is not less than the average cross-validated individual fit error, confirmed the gain achieved when using estimates $\hat{\theta }$ obtained from a separate training population (*p* < 0.0001).

### C. Impact on kinetic modeling

AIF estimation is of interest in the context where there is calculation of kinetic parameters. Impact of the proposed PBPM approach on parametric imaging was evaluated in that sense via Monte Carlo simulation of H_2_O- and FDG-PET time activity curves generated from one-tissue compartment (1C) and two-tissue compartment (2C) models respectively [4], [45]-[48], where

(6) $$\frac{dC_{1}(t)}{dt}=K_{1}C_{p}(t)-(k_{2}+k_{3})C_{1}(t)+k_{4}C_{2}(t)$$

(7) $$\frac{dC_{2}(t)}{dt}=k_{3}C_{1}(t)-k_{4}C_{2}(t)$$

(with *k*_3_ = *k*_4_ = 0 in the 1C model) describe exchanges between arterial plasma *C*_*p*_ and the first and second tissue compartments *C*_1_ and *C*_2_. Details on the modeling and implementation of this system for this analysis are provided in Appendix B. A set of template H_2_O time activity curves $\{z_{{}_{t},\text{true}}{\}}_{i=1}^{N}$ were created by evaluating the 1C model with each of the *N* = 39 clinical arterial samples from the H_2_O dataset described in Section III-A and with true parameter values *K*_1_ = 0.2 mL · cm^−3^ · min^−1^, *k*_2_ = 0.8 min^−1^, *V*_*B*_ = 0.05 mL · cm^−3^, Δ = 0.25 s (found relevant in other studies [49] in modeling tissue activity around the aorta in tumor blood flow studies). Similarly, a set of template FDG time activity curves $\{z_{{}_{t},\text{true}}{\}}_{i=1}^{N}$ were obtained by evaluating the 2C model with each of the *N* = 91 clinical arterial samples from the FDG dataset of Section III-A and with true parameter values *K*_1_ = 0.102 mL · cm^−3^ · min^−1^; *k*_2_ = 0.13 min^−1^; *k*_3_ = 0.062 min^−1^; *k*_4_ = 0.0068 min^−1^; *V*_*B*_ = 0.04 mL · cm^−3^; Δ = 0.2667 s (found relevant in other studies [50] for modeling of grey matter activity in cerebral MRglu studies). This process yielded a set of respectively 39 and 91 unique template uptake curves. These templates were normalised to 1 so estimation of a scale factor was not required in this experiment. Finally, simulated uptake curves were obtained for each scenario by adding Gaussian noise with standard deviation $\sigma =\phi \sqrt{z_{{}_{t},\text{true}}}$ with *ϕ* = 0.001 for H_2_O and *ϕ* = 0.04 for FDG, to 10 replicates of each of these template curves. This yielded respectively 390 and 910 simulated time activity curves for these two settings.

Estimates of flow (*K*_1_), rate *k*_2_, and distribution volume *V*_*D*_ = *K*_1_/*k*_2_ for the H_2_O scenario, and of flux (*K*_*i*_ = *K*_1_*k*_3_/(*k*_2_ + *k*_3_)), distribution volume (*V_D_* = *K_i_*/*k*_3_) and rates *K*_1_, *k*_2_ and *k*_3_ for the FDG scenario, were compared across the range of 1C and 2C model fits obtained using AIF estimates from BCM, TE, CTE and PBPM for the H_2_O- and FDG-PET cohorts. Note that in the FDG case here, quantity *V*_*D*_ [51], [52] differs from the distribution volume *V*_*t*_ = *K*_1_/*k*_2_(1 + *k*_3_/*k*_4_) of total ligand uptake in tissue relative to total concentration of ligand in plasma typically of interest for 2-TC models, since we assume *k*_4_ = 0 [53]. For FDG, the distribution of non-displaceable compartment relative to total concentration of ligand in plasma *V*_*nd*_ = *K*_1_/*k*_2_ is also reported on. (In the case of water, these quantities are equivalent.) Delay was held constant for the purpose of this analysis, and the compartment models were optimized with respect to (*K*_1_, *k*_2_, *k*_3_, *V*_*B*_) (with *k*_3_ = *k*_4_ = 0 in the 1C model, and *k*_4_ = 0 in the 2C model) via nonlinear estimation.

**Fig. 12.**
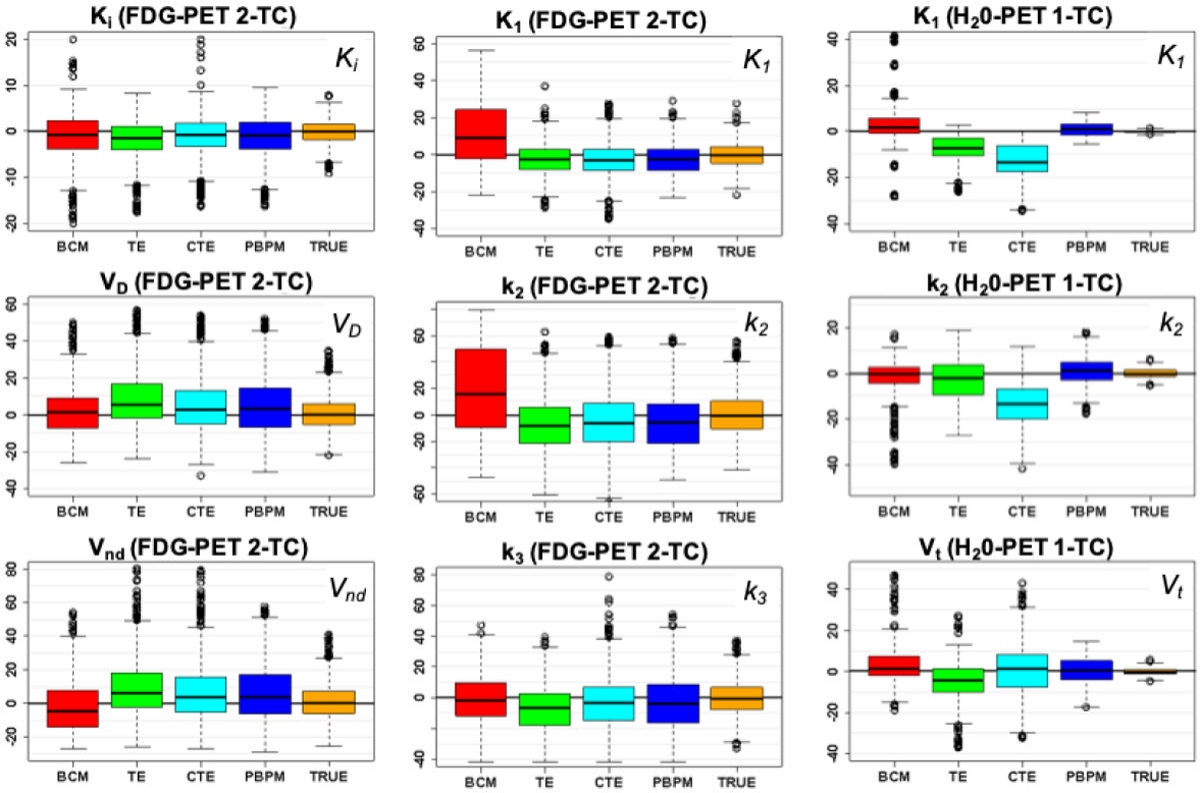
Distributions of percentage errors in estimates of flux ***K***_***i***_ (top left), distribution volume ***V***_***D***_ (centre left), ***V***_***nd***_ (bottom left), ***K***_**1**_ (centre, top), ***k***_**2**_ (center), and ***k***_**3**_ (centre, bottom) parameters from 2C modeling of simulated FDG uptake data, and of flow ***K***_**1**_ (top right), ***k***_**2**_ (center right) and ***V***_***t***_ (bottom right) parameters from 1C modeling of simulated H_**2**_O uptake data, using respectively BCM, TE, CTE, PBPm AIF estimates, or the true AIF used in simulating the template curves.

Figure 12 shows the distributions of percentage errors in estimating these parameters using varying AIF estimates, and Table III provides the p-values of corresponding one-tailed Wilcoxon (Mann-Whitney) tests to assess bias from these distributions, under the null hypothesis of a strictly lower bias for the alternative model, compared to PBPM (i.e. *H*_0_ : *Err*_*PBPM*_ ≥ *Err*_*other*_, after adjustment of error signs where appropriate for the comparison). In complement, “mirror” tests assessing *H*_0_ : *Err*_*PBPM*_ ≤ *Err*_*other*_ indicated that PBPM bias was significantly greater than BCM bias for estimation of *V*_*nd*_ and rate *k*_3_ in the FDG setting (*p* < 0.005 for both). No other such tests yielded significance. In particular, TE and CTE did not yield significantly lower bias compared to PBPM in any scenario.

In summary, the PBPM model is a viable option for evaluation of physiological parameters in the FDG and H_2_O settings, where it led to either statistically comparable or significantly reduced bias in every scenario for all kinetic parameters, with the only exception of BCM for estimation of *V*_*nd*_ and *k*_3_ in FDG. These results illustrate its competitiveness and reliability for kinetic analysis across different tracers, against the most effective methods of reference.

## V. Discussion

This paper introduced a novel population-based projection model (PBPM) that combines strategies used in both image-derived input function (IDIF) and population-based input function (PBIF) approaches in order to achieve flexible and reliable AIF estimation throughout the whole time course, by fitting a mixture of population-informed uptake patterns to individual patient imaging data. In this sense this methodology can be seen as the projection of individual patient PET tracer kinetics characteristics onto a basis of population uptake Using a few population-based components, the proposed method can incorporate knowledge of the injection duration into the model fit, thus not requiring the injection protocol to be the same in all PET studies, unlike common PBIF methods.

A comparative analysis was carried out that included two reference models, namely the tri-exponential (TE) and convolved tri-exponential (TCE) models, and a whole-body blood circulation model (BCM) as references for FDG-, H_2_O- and FLT-PET datasets, as well as simulated datasets obtained from resampling of the clinical information. Results demonstrated that the proposed PBPM has overall competitive or lower AIF estimation error and greater adaptability to different tracers compared to the other three methods, and illustrated its consistency at varying noise levels. Application of the proposed approach to parametric imaging, using one- and two-tissue compartment kinetic modeling scenarios, was also evaluated. In this experiment, the PBPM method overall yielded appropriate characterisation of kinetic rates as well as flow, flux and distribution volume, and yielded either comparable or significantly improved bias over the other models (except for two cases where alternate BCM yielded statistically significant improvement over PBPM), showing improved adaptivity to different tracers.

A natural end-goal is to apply AIF modeling to image-derived data, either because clinical sampling is not available or to facilitate non-invasive evaluation. As part of the technical contributions of this paper, regularization of the model should confer robustness against increased noise levels typically found in imaged blood-pool data. A region of interest from PET imaging of the left ventricle could be used in this sense, as the contrast of concentration of tracer from ventricles and their surrounding muscle is usually distinct. Follow-on work is planned around future access to datasets that combine sampling and imaging data to carry out such an evaluation. This will also yield the opportunity to evaluate the proposed PBPM method compared to IDIF techniques used commonly with major PET tracers.

Where only individual venous blood samples and no reliable arterial blood information are available, the proposed technique may still be considered, for example after conversion of the venous signals into arterial scales. Some venous input function (VIF) approaches were proposed that apply an arteriovenous transform scheme to estimate AIF, for example in ^11^C-labelled studies [54].

The requirement for scale factor estimation, discussed in Section II-D, impacts all modeling techniques, including the proposed PBPM strategy, but it is not yet clear how. Analysing this impact will be the scope of follow-on work.

A critical minimum number of PET studies required to use the proposed PBPM technique with future studies remains to be determined and could vary with the PET radiotracer used. Numerical experiments demonstrated robustness of this model in this aspect, with stable model fit errors for even very few population studies.

This study has not discriminated the data in terms of healthy and cancer subjects, nor in terms of disease types. Although we do not anticipate significant differences in AIF profiles across such strata, using imaged data may introduce differences in PBPM estimates of AIF profiles. Further analyses would be required to explore this.

## VI. Conclusion

We proposed a statistical model of the arterial input function of a patient that leverages arterial uptake profile information at both the cohort and subject levels. Results from simulations demonstrated numerical stability and consistency of the approach at varying noise levels. Improvement in accuracy of the AIF representation over reference AIF models was assessed on three clinical cohorts, each imaged with one of three routinely used PET tracers (namely, FDG, H_2_O and FLT). The proposed model either outperformed or remained competitive against three reference techniques, in terms of AIF estimation RMSE, for these tracers. Experiments further demonstrated that this representation of the AIF is viable for kinetic analysis of physiological parameters such as flow, flux and distribution volume from one- and two-tissue compartment models. Overall the proposed model yielded statistically comparable or significantly lower bias against the three alternate models for determination of kinetics from typical FDG template data in almost all comparisons. The proposed methodology can be incorporated in either population-based or image-derived input function estimation frameworks; the latter will be the scope of follow-on work assessing this model for non-invasive AIF estimation.

## Figures and Tables

**TABLE I:** Top section: patient information in mean (sd) of 105 FDG, 39 H_2_O and 32 FLT studies (only 101 Weight and Height for FDG studies). Bottom section: median sampling durations and median numbers of time points in the sampling frames per tracer.

Tracer	FDG	H_2_O	FLT
Injection Dose (mCi)	8.92 (1.48)	22.72 (20.92)	4.43 (0.59)
Gender	37F; 68M	15F; 24M	7F; 25M
Weight (kg)	79.1 (15.6)	76.1 (14.4)	82.4 (17.3)
Height (cm)	174.7 (9.6)	175.8 (8.3)	175.6 (9.5)
Peak (mCi)	1.87 (0.57)	13.57 (15.03)	0.77 (0.47)
Number of sampling points	26	30	27
Sampling duration (min)	90	4.917	110

**TABLE II:** One-sided Mann-Whitney test p-values for the cross-validated BCM, TE, CTE and PBPM MSEs of Fig. 9. The alternative hypotheses ***H***_***A***_ for these tests are provided to assist in interpreting the test outputs. Here ***H***_***A***_ is accepted at the 5% significance level for p-values lower than 0.05 (significant results in bold).

Model Reference	FDG	H_2_O	FLT
MSE: mean (sd)			
BCM	0.0116 (0.0094)	0.0086 (0.0069)	0.0127 (0.0072)
TE	0.0073 (0.0047)	0.0099 (0.0040)	0.0066 (0.0035)
CTE	0.0073 (0.0047)	0.0169 (0.0080)	0.0112 (0.0055)
PBPM	0.0063 (0.0045)	0.0073 (0.0039)	0.0070 (0.0041)
Mann-Whitney p-value			
H_A_: MSE_BCM_>MSE_PBPM_	**< 0.001**	0.312	**0.002**
H_A_: MSE_TE_>MSE_PBPM_	**0.002**	**0.004**	0.512
H_A_: MSE_CTE_>MSE_PBPM_	**< 0.001**	**< 0.001**	**0.010**

**TABLE III:** One-sided Mann-Whitney test p-values for the percentage errors in parameter estimates of Fig. 12. Cases where the alternative hypothesis ***H***_***A***_ that the percentage error of PBPM is strictly lower than that of the model it is compared to for that test (after a sign adjustment where required) is accepted at the 5% significance level are indicated in bold.

	*K_i_* (FDG)	*K*_1_ (FDG)	*K*_1_ (H_2_O)
BCM	**0.0317**	< **0.0001**	**0.0001**
TE	< **0.0001**	0.7392	< **0.0001**
CTE	0.1417	0.5621	< **0.0001**
	*V_D_* (FDG)	*k*_2_ (FDG)	*k*_2_ (H_2_O)
BCM	< **0.0001**	< **0.0001**	0.4665
TE	**0.0263**	**0.0220**	**0.0074**
CTE	< **0.0001**	0.5170	< **0.0001**
	*V_nd_* (FDG)	*k*_3_ (FDG)	*V_t_* (H_2_O)
BCM	0.9997	0.9954	**0.0006**
TE	**0.0002**	< **0.0001**	< **0.0001**
CTE	0.4531	0.4974	0.2311

## Footnotes

1 e.g. if using small and/or noisy PET imaging data for AIF estimation

## Appendix

Here we provide technical details on the analyses carried out in the main paper for model selection, simulation-based validation studies, and implementation of the one- and two-tissue compartment models used for kinetic analysis.

### A. Validation study on model-derived template

In the first analysis of Section III-C.2, three mixture components were fit to the FDG-PET imaging cohort to obtain estimates of the Gamma and exponential parameters with parameters set as follows:

$$\;\hat{\alpha }=2.1784,\hat{\beta }=7.8741,\;{\hat{\phi }}_{1}=88.3992,{\hat{\phi }}_{2}=0.1488,{\hat{\phi }}_{3}=0.0088.$$

To simulate individual AIF curves, the linear mixing parameters were generated randomly via an acceptance-rejection algorithm, drawing values from a Normal distribution so that $\pi_{i}\;∼\;N({\bar{\pi }}_{i},\sigma (\pi_{i}{)}^{2})$, *i* = 1, 2, and enforcing some *ad hoc* constraints. A value for *π*_3_ was then set given *π*_1_ and *π*_2_ to ensure that $\sum_{i=1}^{3}\pi_{i}\geq 1$, and the simulated time courses were defined as

(8)
$$C_{p}^{tem}(t_{i})=\pi_{1}C_{1}(t_{i}|\theta )+\pi_{2}C_{2}(t_{i}|\theta )+\pi_{3}C_{3}(t_{i}|\theta )$$

The following steps were taken to simulate the AIF curves:

Step 1 - Generate random component curves *C*_1_(*t_i_*∣*θ*), *C*_2_(*t*_*i*_∣*θ*) and *C*_3_(*t*_*i*_∣*θ*) using the nonlinear parameter values given above;

Step 2 - Generate 0.4 < *π*_1_ < 0.8 and 0.08 < *π*_2_ < 0.35 via acceptance-rejection;

Step 3 - Generate $\pi_{3}=\frac{1-max\{\pi_{1}C_{1}(t_{i}|\theta )+\pi_{2}C_{2}(t_{i}|\theta )\}}{max\{C_{3}(t_{i}|\theta )\}}$;

Step 4 - Test that *π*_3_ satisfies

(9)
$$0.17<\pi_{3}<0.37$$

(10)
$$0.99<\pi_{1}+\pi_{1}+\pi_{3}<1.15$$

and repeat Steps 2–4 until the above constraints are satisfied, to ensure adequate mixing coefficient values.

In the second analysis of Section III-C.2, sets of 400 simulated curves were generated using the same process at varying noise levels termed *high, mid-high, medium, mid-low* and *low*

(11)
$$C_{P}^{sim}(t_{i})=C_{p}^{tem}(t_{i})+\epsilon (\cdot ,t_{i})$$

where *ϵ*(*medium*) ~ *N*(0, .05) when *t*_*i*_ < 1.5 min and *ϵ*(*medium*) ~ *N*(0, .01) when *t*_*i*_ ≥ 1.5 min for level *medium*. *ϵ*(*high*), *ϵ*(*mid-high*), *ϵ*(*mid-low*) and *ϵ*(*low*) are 3, $\frac{3}{2}$, $\frac{2}{3}$ and $\frac{1}{3}$ times of *ϵ*(*medium*), respectively.

### B. Evaluation of compartment models

Here we provide details on the parametric modeling approach used for kinetic analysis in Section IV-C. The arterial plasma concentration of the tracer is the input for kinetic models. Direct measurement of plasma concentration may not always be feasible, but if the arterial whole blood activity is known (or estimated), it may be used to infer the plasma concentration [29], [55]. Implementation of the one- and two-tissue compartment models given concentration in plasma *C*_*P*_(*t*) involves computation of the concentration in tissue *C*_*T*_(*t*), represented as the following convolution:

$$C_{T}(t)=(\varphi_{1}e^{-\theta_{1}t}+\varphi_{2}e^{-\theta_{2}t})\;⊗\;C_{P}(t)$$

This concentration *C*_*T*_(*t*) can be seen as the sum of concentrations in compartments 1 and 2, i.e. *C*_*T*_(*t*) = *C*_1_(*t*) + *C*_2_(*t*). Exchanges between arterial plasma *C*_*p*_ and the first and second tissue compartments *C*_1_ and *C*_2_ are described respectively as follows (with *k*_3_ = *k*_4_ = 0 in the 1C model) [4], [9], [48], [56]:

(12)
$$\frac{dC_{1}(t)}{dt}=K_{1}C_{p}(t)-(k_{2}+k_{3})C_{1}(t)+k_{4}C_{2}(t)$$

(13)
$$\frac{dC_{2}(t)}{dt}=k_{3}C_{1}(t)-k_{4}C_{2}(t).$$

The compartment-specific contributions are derived from Eqn. (12) and Eqn. (13) respectively as follows ([9], [56]):

$$C_{1}(t)=\frac{K_{1}}{\theta_{2}-\theta_{1}}\left((k_{4}-\theta_{1})e^{-\theta_{1}t}+(\theta_{2}-k_{4})e^{-\theta_{2}t}\right)⊗C_{p}(t)\;C_{2}(t)=\frac{K_{1}k_{3}}{\theta_{2}-\theta_{1}}\left(e^{-\theta_{1}t}-e^{-\theta_{2}t}\right)⊗C_{P}(t)$$

Specifically, we can use

$$\Delta =\sqrt{(k_{2}+k_{3}+k_{4}{)}^{2}-4k_{2}k_{4}}\;\theta_{1}=\frac{k_{2}+k_{3}+k_{4}+\Delta }{2}\;\theta_{2}=\frac{k_{2}+k_{3}+k_{4}-\Delta }{2}\;\varphi_{1}=K_{1}\frac{\theta_{1}-k_{3}-k_{4}}{\Delta }\;\varphi_{2}=-K_{1}\frac{\theta_{2}-k_{3}-k_{4}}{\Delta }$$

to evaluate the model for given (*K*_1_, *k*_2_, *k*_3_, *k*_4_) values. For the one-tissue compartment model, we simply have *k*_3_ = *k*_4_ = 0.
